# The importance of age, sex and place in understanding socioeconomic inequalities in allostatic load: Evidence from the Scottish Health Survey (2008–2011)

**DOI:** 10.1186/s12889-016-2796-4

**Published:** 2016-02-09

**Authors:** Tony Robertson, Eleanor Watts

**Affiliations:** 1 School of Health Sciences, University of Stirling, Stirling, FK9 4LA UK; 2 Cancer Epidemiology Unit, University of Oxford, Oxford, OX3 7LF UK

**Keywords:** Allostatic load, Socioeconomic Position, Education, Health Inequalities

## Abstract

**Background:**

Given the broad spectrum of health and wellbeing outcomes that are patterned by socioeconomic position (SEP), it has been suggested that there may be common biological pathways linking SEP and health. Allostatic load is one such pathway, which aims to measure cumulative burden/dysregulation across multiple physiological systems. This study aimed to determine the contextual and demographic factors (age, sex and place) that may be important in better understanding the links between lower SEP and higher allostatic load.

**Methods:**

Data were from a nationally representative sample of adults (18+): the Scottish Health Survey (2008–2011). Higher SEP (‘1’) was defined as having ‘Higher’-level, secondary school qualifications versus having lower level or no qualifications (‘0’). For allostatic load, a range of 10 biomarkers across the cardiovascular, metabolic and immune systems were used. Respondents were scored “1” for each biomarker that fell into the highest quartile of risk. Linear regressions were run in STATA, including SEP, age (continuous and as a 7-category variable), sex (male/female), urbanity (a 5-category variable ranging from primary cities to remote rural areas) and geographical location (based on 10 area-level healthboards). Interactions between SEP and each predictor, as well as stratified analyses, were tested.

**Results:**

Lower SEP was associated with higher allostatic load even after adjusting for age, sex and place (*b* = −0.631, 95 % CI −0.795, −0.389, *p* < 0.001). There was no significant effect moderation between SEP and age, sex or place. Stratified analysis did show that the inequality identified in the baseline models widened with age, becoming significant at ages 35–44, before narrowing at older ages (75+). There was no difference by sex, but more mixed findings with regards place (urbanity or geographical location), with a mix of significant and non-significant results by SEP that did not appear to follow any pattern.

**Conclusions:**

Inequalities in allostatic load by educational attainment, as a measure of SEP, are consistent with age, sex and place. However, these stratified analyses showed that these inequalities did widen with age, before narrowing in later life, matching the patterns seen with other objective and subjective health measures. However, effect moderation analysis did not support evidence of a statistically significant interaction between age and SEP. Context remains an important feature in understanding and potentially addressing inequalities, although may be less of an issue in terms of physiological burden.

**Electronic supplementary material:**

The online version of this article (doi:10.1186/s12889-016-2796-4) contains supplementary material, which is available to authorized users.

## Background

Throughout the world individuals with lower socioeconomic position (SEP) experience lower levels of health and reduced life expectancy [1]. This trend is continuous and exhibits no threshold limit; even those of relatively high SEP experience reduced levels of health in comparison to those with a slightly greater SEP [2], known as the social gradient in health. Socioeconomic differences exist for almost every major contemporary and historical cause of mortality and morbidity, highly suggestive of a common physiological pathway [3, 4]. Mechanistically, chronic and/or high levels of stress evoke over-activation of the hypothalamic-pituitary-adrenal (HPA) and sympathetic-adrenal-medullary (SAM) axes and prolong the secretion of neuroendocrine measures (e.g. cortisol) as primary mediators, before later effects on secondary (e.g. blood pressure) and tertiary (e.g. cellular ageing) outcomes [5]. Sustained exposure results in dysregulation and desensitisation to the stress response (reviewed by McEwen, 2002 [6]). These exposures manifest in a reduced capacity to adapt [7], inducing a recalibration process to restore the normative environment, known as allostasis [8]. Although allostasis is beneficial in the short-term, chronic, high levels result in cumulative damage, known as allostatic load [9, 10]. The measure used most often is a simple count score of the number of biomarkers that fall into the highest quartile of risk of the sample distribution [11]. Additional common constructs include using clinical cut-offs or z-scores, although there is no agreed ‘preferred’ construct currently [12]. High allostatic load, which is typically measured using a number of the body’s physiological biomarkers (primarily constructed using secondary outcomes of the stress pathway, as described above) as indicators of regulation/dysregulation [13], is predictive of an increased risk of a number of adverse medical conditions [14–16], and all-cause mortality [13]. Subsequent reduction in allostatic load is also associated with a reduction in risk of dying [17].

A growing literature has identified a consistent association between lower SEP and higher allostatic load across multiple cohorts and population samples [18–24]. However, less attention has been focused on how this association differs within populations given different individual characteristics such as age, sex and place (urbanity and geographic location. Understanding how the relationship between SEP and allostatic load differs according to such individual characteristics across the lifecourse will be an important step in ensuring that the allostatic load concept can be used effectively across the entire population and in developing policies and programmes to reduce inequalities that are effective across these characteristics. The aim of this study was to acquire a better understanding of the social patterning of allostatic load given these demographic/contextual factors using data from a large, nationally representative health survey, an important consideration in the development of more effective and targeted interventions to equitably reduce allostatic load and health inequalities in the general population. We hypothesised that allostatic load would be higher in those with lower SEP, with these inequalities widening with age (given the accumulated nature of the SEP-allostatic load relationship [22]) and then narrowing at older ages (matching results for other health outcomes). We did not predict that the SEP-allostatic load relationship would change between men and women or by urbanity. With geographic location, we hypothesised that Greater Glasgow and Clyde would have the widest inequalities, given the city’s well-publicised poor health record in relation to the rest of Scotland, the UK and Europe [25–28].

## Methods

### Study sample

**Fig. 1:**
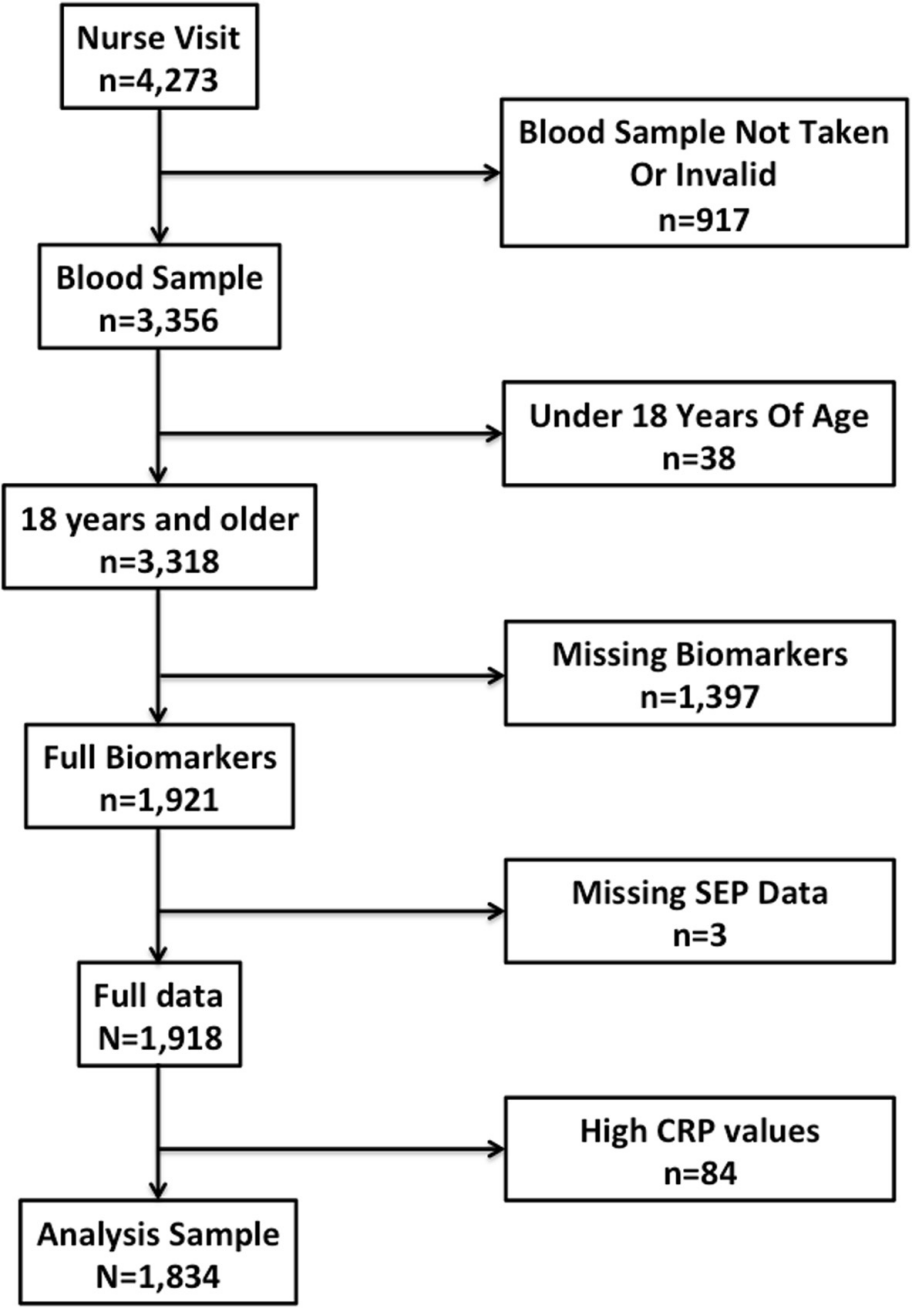
Details of the original and analysis sample sizes

This study used data from the Scottish Health Survey (SHeS) to examine the effect of sex, age and place on the association between allostatic load and SEP. This data is freely available from UK Data Service (http://ukdataservice.ac.uk/). The combined 2008–11 datasets were used, which possess relatively uniform design and measurements to maximise sample size. Only adults (aged ≥18 years) are included in this study as clinical assessments were not carried out on children. The SHeS is an annual cross-sectional study (since 2008, with previous waves in 1995, 1998 and 2003), which provides information of the Scottish population’s health for children and adults residing within private households. Respondents were randomly selected from private households and with up to two adults and two children from each household eligible to participate. All participants were interviewed with in-depth questions covering general perceived health, lifestyle, income and health behaviours. A random selection of adults within the sample (*n* = 4,273, approximately 11 % of total) had a second visit from a nurse for urine, saliva and blood samples alongside blood pressure readings and anthropometric measurements. Consent to take blood within this selected sample was 86 % (*n* = 3,580), although only 3,356 blood samples were successfully collected (Fig. 1). Of these, 38 were excluded for being under 18 years of age (see Methods > Socioeconomic Position for the reasoning), leaving 3,318. Excluding participants who did not have measurements for all biomarkers for the full allostatic load score reduced the sample size to 1,921. A more detailed account of the surveillance methodology can be found elsewhere [29]. Ethical approval for the study was granted by the Research Committee for Wales (08/MRE09/62).

### Allostatic load

Allostatic load is measured via surrogate measures using a range of biomarkers. These were selected *a priori* on the basis of previous research and data availability (i.e. neuroendocrine markers were not available) [12, 30]. Ten biomarkers met the inclusion criteria, these represented a range of physiological systems: **cardiovascular** (pulse rate, systolic blood pressure (SBP) and diastolic blood pressure (DBP)); **metabolic** (body mass index (BMI), waist-to-hip ratio (WHR), high-density lipoprotein (HDL) cholesterol, total cholesterol and glycated haemoglobin (HbA_1C_)) and **immune** (fibrinogen and C - reactive protein (CRP)). Although each biomarker may not contribute to allostatic load equally, they were given equal weight as prior research indicates that an equally weighted algorithm provides an adequately predictive estimate of the true effect of allostatic load [17]. SBP and DBP, pulse rate, WHR and BMI were obtained from physical measurements. HbA_1C_, fibrinogen, CRP and cholesterol were obtained from blood samples. Details about collection of these measures is available in the SHeS Technical Report [29].

Those prescribed blood pressure lowering medication at the time of the survey were adjusted for by adding 10 mmHg and 5 mmHg to their respective SBP and DBP [31]. Those taking diabetes medication had 1 % added to their HbA_1C_ values [32]. The use of statins resulted in the addition of 21.24 mg/dL (1.18 mmol/l) to total cholesterol levels [33], and participants on diuretics had their total cholesterol reduced by 4 %, whilst beta-blockers resulted in the addition of 10 % to HDL cholesterol [34]. Medication effect was manually entered into each biomarker because the allostatic load outcome is entered into the regression model as a singular value and ensures the greatest possible prediction accuracy in incorporating the effects of medication, without risking the incorporation of too many covariates (discussed by Pocock et al., 2002 [35]). Sensitivity analysis using medications as confounders revealed no significant alterations to the main results reported.

Each individual biomarker’s empirical threshold risk was calculated by ranking each biomarker relative to the other respondents. The highest 25th percentile of the population was selected for each biomarker. With the exception of HDL cholesterol, which used the lowest 25th percentile, as this is a strong inverse predictor of cardiovascular disease [36, 37]. The respondents were scored “1” for each biomarker in the high risk quartile, which was computed into an equally weighted allostatic load score out of a maximum of 10, following the standard methodology [9, 18, 38, 39], enabling identification of relative dysregulation within the population.

### Socioeconomic position

Education level (qualifications attained) was used as an indicator of SEP as it represents a long-term and stable indicator of SEP [40]. Qualifications were transformed from sextile (ranging from no qualifications to degree level) to a binary measure. Higher SEP was classified as possessing the equivalent of ‘Highers’ and above, whilst lower SEP was defined as those possessing up to ‘Standard Grade’ qualifications [41, 42]. Standard Grades were the main type of qualification in schools for 15 and 16 year olds in Scotland, and were generally taken over two years in third and fourth year at secondary school (up until 2013 when they were replaced with ‘National Qualifications’). Highers are suitable for learners who have progressed from Standard Grade, typically taken at age 17 during fifth year at secondary school and are the typical requirement for entry into university education. Those under the age of 18 were excluded to avoid misclassification due to being too young to have sat the Higher grade exams (typically taken at age 17) (*n* = 38).

### Age, sex and place

Age was transformed into an age^2^ variable to account for non-linear effects [39, 43]. Sex was a binary variable (male and female). Two place variables were used. First, an urban/rural measures was created using 6 categories, ranging from primary city (population >125,000) to remote rural location. Towns and rural locations were stratified into accessible and remote (methodology in National Statistics Publication for Scotland, 2011 [44]), because those of working ages tend to live in accessible towns and villages surrounding cities and commute into the primary city for work, whilst less-accessible locations tend to exhibit less wealth [45], so it was hypothesised these areas may have different environments and thus stratification will reduce confounding. The primary city group was selected as the reference variable as its mean allostatic load score $\left(\overline{x}=2.42\right)$ was similar to the overall sample mean $\left(\overline{x}=2.55\right)$ and encompassed the greatest population (*n* = 657), and so was deemed adequately representative of the sample. A second variable looked at geographic location, based on National Health Service health board. In Scotland, there are 14 regional health boards, reduced to 10 given low sample sizes. This resulted in Borders and Dumfries and Galloway being combined, as well as the Islands being combined with the Highlands. These groupings remained geographically relevant given the close proximity of the areas (Borders and Dumfries & Galloway) and/or typical groupings used in other local authority settings (Highlands and Islands). Lothian health board was selected as the reference category given its mean allostatic load score $\left(\overline{x}=2.55\right)$ was similar to the overall sample mean $\left(\overline{x}=2.42\right)$ and encompassed the greatest population (*n* = 330).

### Statistical analysis

All analyses were carried out using linear regression in Stata version 11. The baseline model assessed the association between SEP and allostatic load only, with no other variables included in the model. This baseline model was then built on by including the variable of interest (age, sex or place) into the model, including an interaction term with SEP to assess effect modification. The dataset provided weights to help account for selection bias between those who were asked to give blood (representative of the whole sample) and those who actually consented to blood draws, thereby making the dataset more representative of the original intended sample by incorporating these weights into the regression model [29]. However, these weights cannot account for differences between the final analysis sample and those who consented to bloods.

### Sensitivity analysis

Sensitivity analysis was also used to evaluate the constructs and assumptions used in the linear regression model, as well as to test the robustness of the results. These tests were decided *a priori.* The effect of changing the threshold of the biomarkers from empirical to clinical was examined [15, 21, 46]. However, this measure does not specifically examine indicators of dysregulation *per se*, rather disease/clinical risk prevalence within a population. These measures do however allow for population comparability, as they do not change based on the population studied and will verify if interaction persists at a clinical level. These cut-offs were defined using standard clinical cut-offs (Additional file 1: Web Table 1), and typically used higher cut-offs than the primary analysis. This changed the maximum allostatic load score to eight (from nine), with only two individuals with this score.

## Results

Of the 3,580 respondents who consented to having bloods and anthropometric measures taken, 1,918 had results for all 10 biomarkers and full data for sex, age, place and SEP and were included in the analysis, although 81 were excluded given high CRP values (>10 mg/dL) (Fig. 1). This sample was 44.3 % male, with ages ranging from 18–94 (mean age = 48.7; SD = 15.8) (Table 1). Considerable socioeconomic diversity was evident; one third of the sample attained at least a University degree, whilst 16.5 % had no qualifications. Mean allostatic load was 2.52 and scores ranged from 0–9 (Table 2). The distribution of scores had a positive skew, with 55 % of individuals having an allostatic load score ≤2. No respondents had an allostatic load of 10.

**Table 1:** Characteristics of participants in analysis and by SEP

Covariate	Total	Low SEP	High SEP
Total	1834 (100 %)	716 (39.0 %)	1118 (61.0 %)
SEX			
Male	812 (44.3 %)	311 (43.4 %)	501 (44.8 %)
Female	1022 (55.7 %)	405 (56.6 %)	617 (55.2 %)
AGE			
18-24	109 (5.9 %)	37 (5.2 %)	72 (6.4 %)
25-34	265 (14.4 %)	40 (5.6 %)	225 (20.1 %)
35-44	398 (21.7 %)	123 (17.2 %)	275 (24.6 %)
45-54	391 (21.3 %)	157 (21.9 %)	234 (20.9 %)
55-64	363 (19.8 %)	163 (22.8 %)	200 (17.9 %)
65-74	197 (10.7 %)	124 (17.3 %)	73 (6.5 %)
75+	111 (6.1 %)	72 (10.1 %)	39 (3.5 %)
Urban/Rural			
Primary City (pop > 125,000)	626 (34.1 %)	216 (30.2 %)	410 (36.7 %)
Urban (pop > 10,000)	530 (28.9 %)	222 (31.0 %)	308 (27.5 %)
Small Accessible town (pop > 3,000)	165 (9.0 %)	75 (10.5 %)	90 (8.1 %)
Small Remote town (pop > 3,000)	74 (4.0 %)	36 (5.0 %)	38 (3.4 %)
Accessible Rural	299 (16.3 %)	103 (14.4 %)	196 (17.5 %)
Remote Rural	140 (7.6 %)	64 (8.9 %)	76 (6.8 %)
Location			
Ayrshire & Arran	115 (6.3 %)	51 (7.1 %)	65 (5.8 %)
Borders, Dumfries & Galloway	114 (6.2 %)	47 (6.6 %)	68 (5.9 %)
Fife	167 (9.1 %)	49 (6.8 %)	124 (10.7 %)
Forth Valley	104 (5.7 %)	42 (5.9 %)	65 (5.6 %)
Grampian	232 (12.6 %)	102 (14.2 %)	137 (11.8 %)
Greater Glasgow & Clyde	299 (16.3 %)	125 (17.5 %)	180 (15.5 %)
Highland & Islands	141 (7.7 %)	59 (8.2 %)	83 (7.2 %)
Lanarkshire	166 (9.1 %)	72 (10.1 %)	95 (8.3 %)
Lothian	312 (17.0 %)	100 (14.0 %)	220 (19.0 %)
Tayside	184 (10.0 %)	69 (9.6 %)	117 (10.1 %)

**Table 2:** Descriptive statistics of individual biomarkers among adults (≥18 years), Scottish Health Survey (*n* = 1,834)

System	Biomarker	High Risk Threshold (Allostatic load score +1)	Clinical risk threshold	Range	Mean Score (Standard Deviation)
*Cardiovascular*	Pulse Rate (bpm)	≥76.3 bpm	≥100 bpm	37.7 − 108.0	69.9 (10.40)
	Systolic Blood Pressure (mmHg)	>138.5 mmHg	≥140 mmHg	87.5 − 218.5	128.5 (17.84)
	Diastolic Blood Pressure (mmHg)	>82.5 mmHg	≥90 mmHg	38.5 − 114.0	75.2 (10.97)
*Metabolic*	BMI (kg/m^2^)	>30.0 kg/m^2^	>30 kg/m^2^	14.0 – 47.0	27.4 (4.64)
	Waist-to-hip ratio (Men)	≥0.93	≥0.94	0.76 − 1.15	0.93 (0.07)
	Waist-to-hip ratio (Women)	≥0.93	≥0.80	0.66 − 1.08	0.83 (0.07)
	HDL cholesterol (mmol/L)	≤1.20 mmol/L	≤1.00 mmol/L	0.5 − 3.3	1.50 (0.40)
	Total cholesterol (mmol/L)	>6.24 mmol/L	>6.20 mmol/L	2.5 − 10.4	5.55 (1.08)
	HbA1c (%)	≥5.80 %	≥5.70 %	4.1 − 13.7	5.57 (0.57)
*Immune*	Fibrinogen (g/L)	≥3.40 g/L	≥4.0 g/L	1.1 − 5.0	3.00 (0.51)
	C-Reactive Protein (mg/L)	≥2.70 mg/L	≥10.0 mg/L	0.1 – 10.8	2.02 (2.02)
*Allostatic load score*		-	-	0 − 9	2.52 (1.95)

Increasing age was associated with higher allostatic load (*b* = 0.051, 95 % CI 0.036, 0.067, *p* < 0.001), representing a 1-unit increase in allostatic load with every 19.4 years. There was no evidence of a non-linear age effect (using an age^2^ and an age^3^ transformation) (age^2^: *b* = −0.080, 95 % CI −0.286, 0.446, *p* = 0.668; age^3^: *b* = −0.001, 95 % CI −0.001, 0.001, *p* = 0.185). Sex differences were present, with female respondents significantly more likely to have lower allostatic load than males (*b* = −0.368, 95 % CI −0.507, −0.169, *p* < 0.001). Urban/rural place of residence was also associated with allostatic load, with residents in all places excluding small remote towns and accessible rural areas showing a higher allostatic load when in compared to primary cities. Compared to Lothian, only respondents from Ayrshire & Arran (*b* = 0.495, 95 % CI 0.058, 0.931, *p* = 0.026) and Fife (*b* = 0.503, 95 % CI 0.061, 0.946, *p* = 0.026) had significantly higher allostatic load scores.

**Fig. 2:**
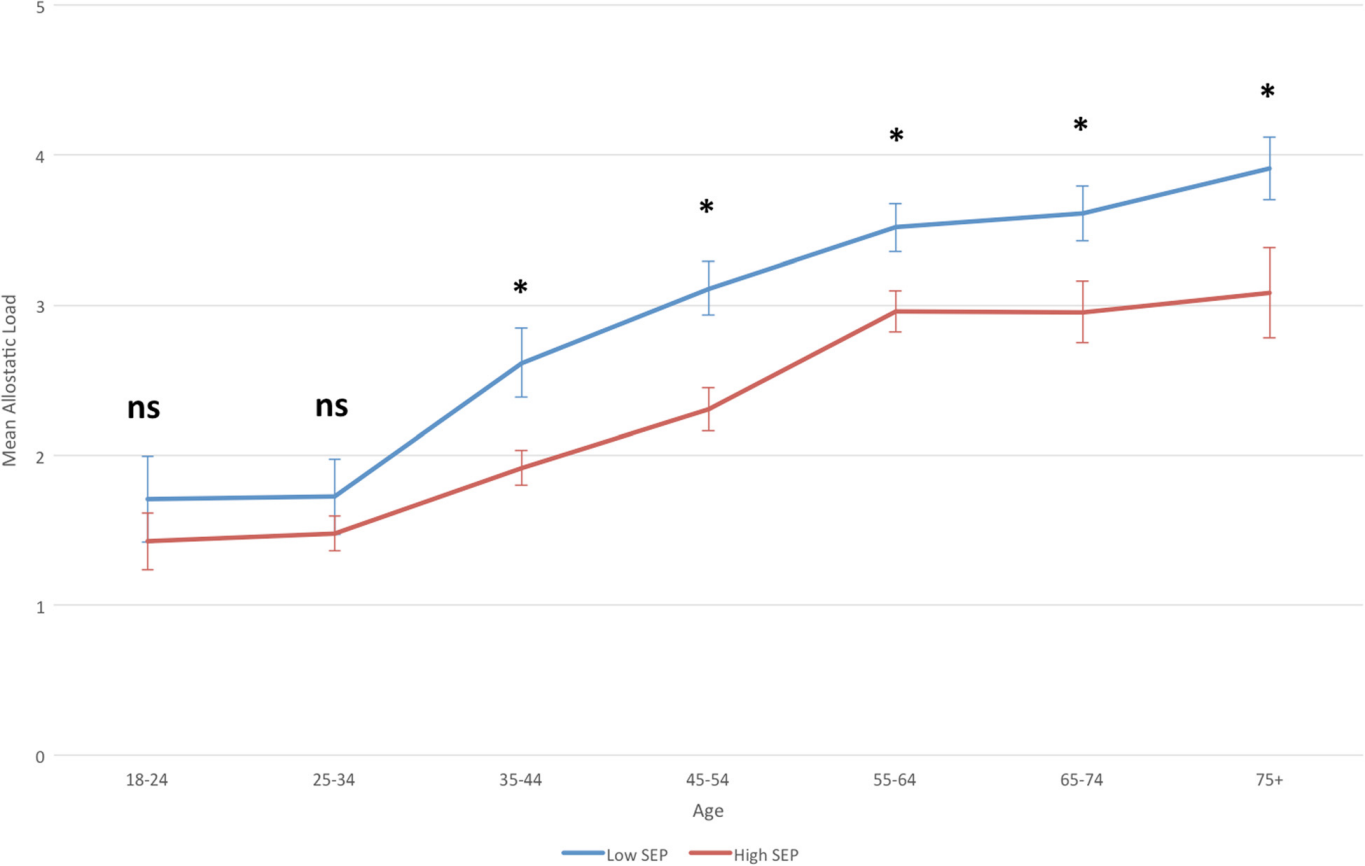
Mean allostatic load (based on quartiles) for men and women, by educational attainment (lower SEP = Standard Grades or below) (with Standard Errors). * = *p* < 0.05

**Fig. 3:**
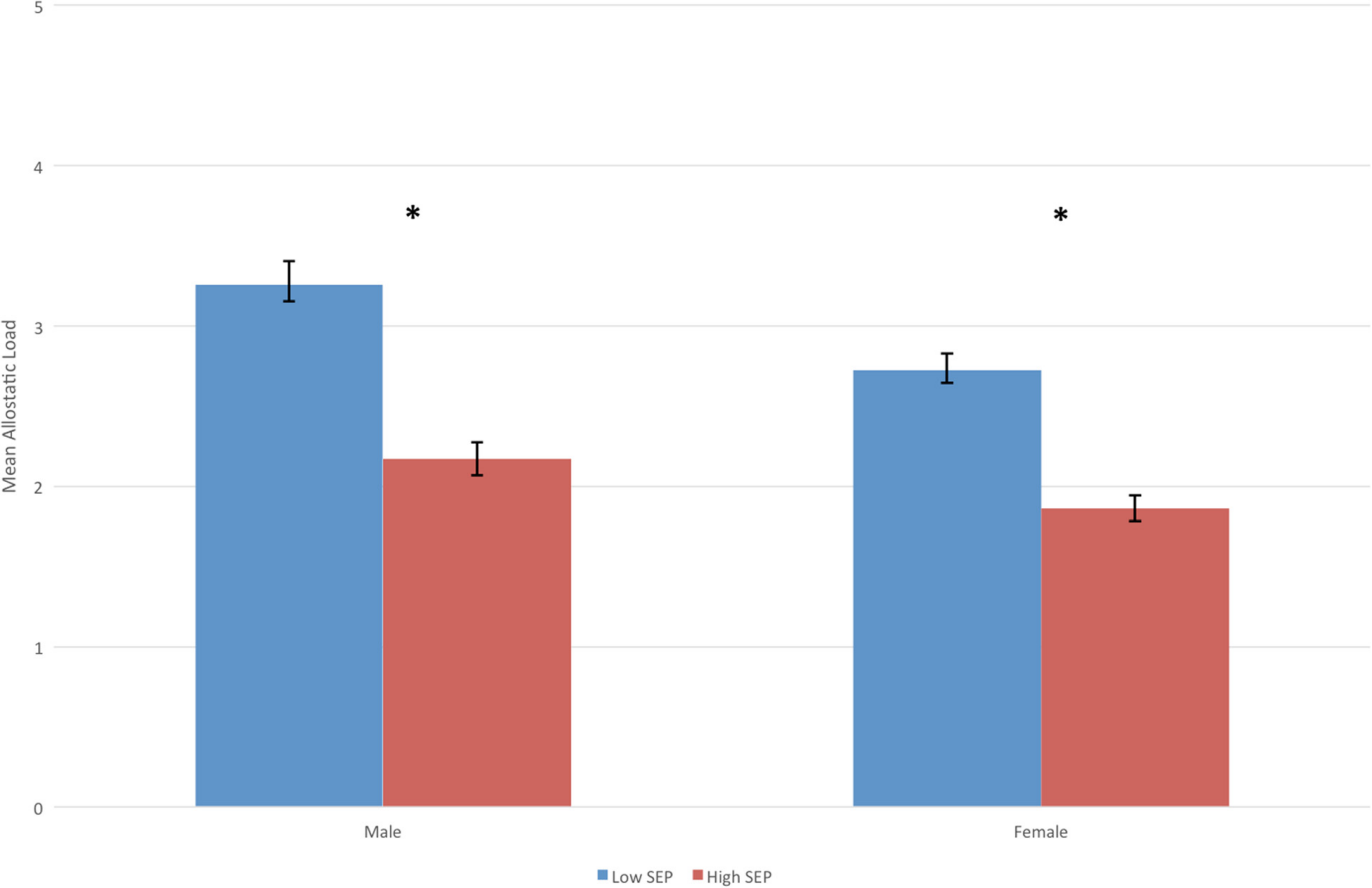
Mean allostatic load (based on quartiles) at different ages, by educational attainment (lower SEP = Standard Grades or below) (with Standard Errors). * = *p* < 0.05

**Fig. 4:**
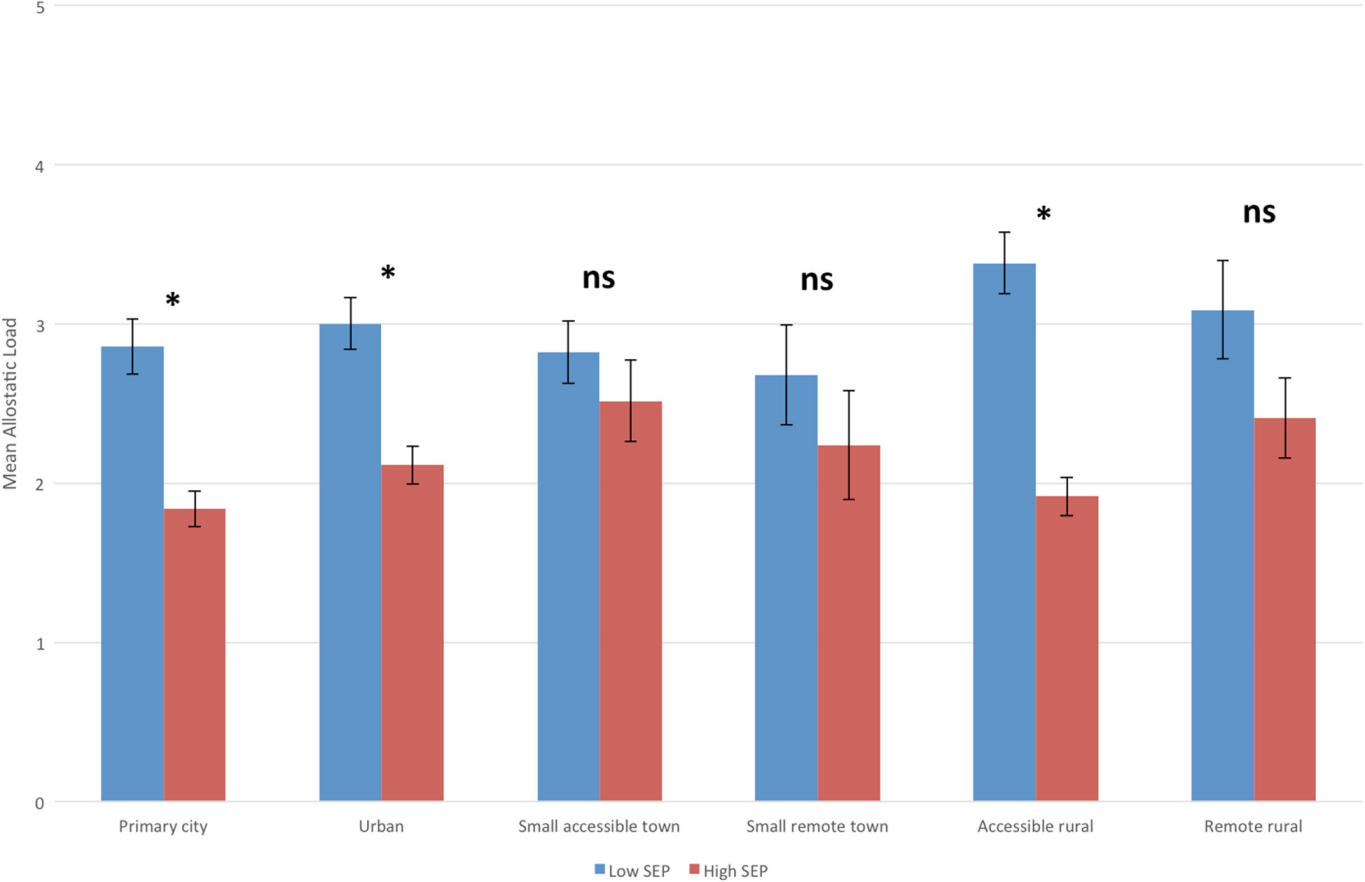
Mean allostatic load (based on quartiles) in different urban and rural locations, by educational attainment (lower SEP = Standard Grades or below) (with Standard Errors). * = *p* < 0.05

**Fig. 5:**
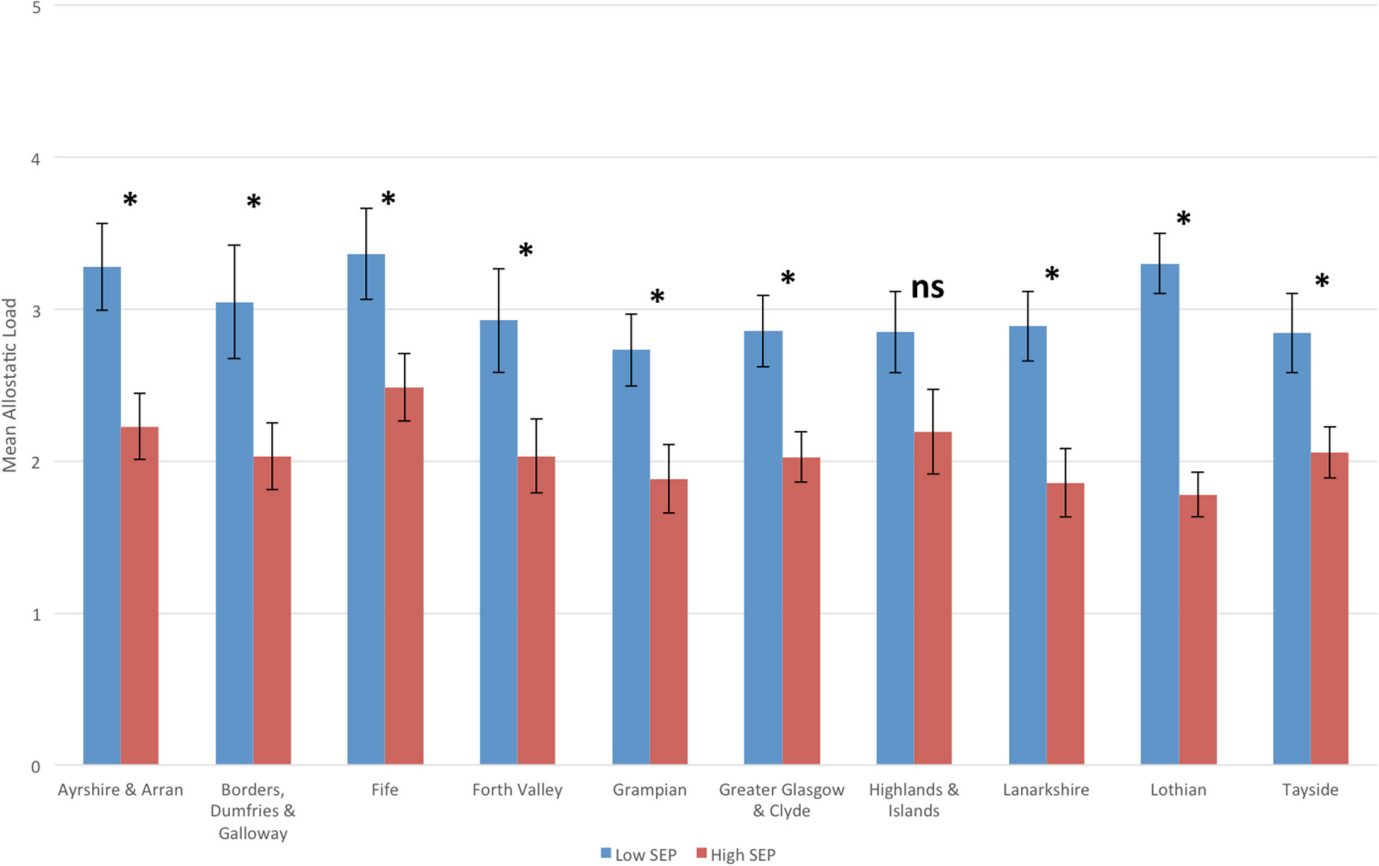
Mean allostatic load (based on quartiles) in different geographical locations, by educational attainment (lower SEP = Standard Grades or below) (with Standard Errors). * = *p* < 0.05

There was a significant inequality in allostatic load by educational attainment, with those with higher SEP having significantly lower allostatic load scores compared to those with lower SEP (*b* = −0.959, 95 % CI −1.179, −0.739 *p* < 0.001). This disparity is the equivalent of a 18.6-year age difference in physiological health, based on the results of the age-allostatic load association described above. This association remained after adjustment for age, but the magnitude of effect was reduced to the equivalent of a 11.6-year difference in physiological burden (*b* = −0.593, 95 % CI −0.798, −0.387, *p* < 0.001). Adjusting for sex, urban/rural location and geographical location did not attenuate the associations (Table 3). Next we tested for any evidence of effect moderation by any of these variables (using interaction terms with SEP). However, for each of the variables, there was no significant interaction effect (*p* > 0.05), with higher allostatic load scores in those with lower SEP consistent across all analyses (Table 4). Although there was no effect moderation, we did examine the SEP inequality by each factor in stratified analyses to visualise these associations in closer detail. For both men and women, there was the expected social gradient in allostatic load, with higher allostatic load with lower SEP (Fig. 2, Table 4). For age, there were significantly higher allostatic load scores in those with lower SEP from ages 35 and above, but this pattern was not seen between 18 and 34 years of age. There was also evidence that this inequality had begun to narrow at older ages (75+) (Fig. 3, Table 5). For urban/rural, the social patterning of allostatic load was only significant in the primary cities, urban environments and accessible rural environments (Fig. 4, Table 5). Finally for geographical location, allostatic load was only socially patterned and statistically significant in 4 of the 10 locations, with the largest inequality seen in Lothian (which includes the city of Edinburgh) (Fig. 5, Table 5).

**Table 3:** Educational disparities in allostatic load (using quartiles), adjusted for age, sex, urban/rural and location

	Beta (Lower vs Higher SEP)	95 % Confidence Intervals	*P*-value
Baseline model (unadjusted)	−0.959	−1.179, −0.739	<0.001
+ Age	−0.593	−0.798, −0.387	<0.001
+ Age + Age^2^	−0.597	−0.802, −0.387	<0.001
+ Age + Age^2^ + Age^3^	−0.599	−0.803, 0.395	<0.001
+ Sex	−0.969	−1.189, −0.749	<0.001
+ Urban/Rural	−0.935	−1.154, −0.715	<0.001
+ Location	−0.969	−1.187, −0.752	<0.001
+ Age, Sex, Urban/Rural and Location	−0.631	−0.795, −0.389	<0.001

*Lower SEP : Lower socioeconomic position (Scottish Standard Grade qualifications and below), which is also the reference category

** Higher SEP: Higher socioeconomic position (Scottish Higher Grade qualifications and above)

**Table 4:** Interaction effects between educational attainment (SEP/socioeconomic position) and age, sex, urban/rural and location

	Beta	95 % Confidence Intervals	*P*-value
Age * SEP	−0.004	−0.015, 0.007	0.471
Age^2^ * SEP	−0.004	−0.457, 0.376	0.848
Age^3^ * SEP	−0.001	−0.001, 0.001	0.618
Sex * SEP	0.221	−0.212, 0.654	0.316
Urban/Rural *SEP	−0.009	−1.133, 0.114	0.881
Location * SEP	−0.027	−1.100, 0.045	0.460

**Table 5:** Educational disparities in allostatic load (using quartiles), stratified by sex, age groups, urban/rural and geographic location

Covariate	Beta (Lower* vs Higher** SEP)	95 % Confidence Intervals	*P*-value
Overall Effect (Unadjusted)	−0.959	−1.179, −0.739	<0.001
SEX			
Male	−1.085	−1.443, −0.726	<0.001
Female	−0.863	−1.119, −0.608	<0.001
AGE			
16-24	−0.281	−0.955, 0.393	0.410
25-34	−0.243	−0.782, 0.296	0.374
35-44	−0.698	−1.193, −0.204	0.006
45-54	−0.806	−1.262, −0.350	0.001
55-64	−0.559	−1.975, −0.144	0.008
65-74	−0.653	−1.188, −0.119	0.017
75+	−0.831	−1.556, −0.106	0.025
Urban/Rural			
Primary City (pop > 125,000)	−1.019	−1.430, −0.607	<0.001
Urban (pop > 10,000)	−0.889	−1.272, −0.506	<0.001
Small Accessible town (pop > 3,000)	−0.309	−0.986, 0.368	0.368
Small Remote town (pop > 3,000)	−0.439	−1.328, 0.449	0.334
Accessible Rural	−1.467	−1.921, −0.012	<0.001
Remote Rural	−0.686	−1.478, 0.117	0.093
Location			
Ayrshire & Arran	−1.049	−1.773, −0.325	0.005
Borders, Dumfries & Galloway	−1.013	−1.829, 0.198	0.016
Fife	−0.875	−1.613, 0.137	0.021
Forth Valley	−0.891	−1.782, 0.001	0.050
Grampian	−0.847	−1.542, −0.152	0.017
Greater Glasgow & Clyde	−0.829	−1.389, −0.269	0.004
Highland & Islands	−0.653	−1.368, 0.062	0.073
Lanarkshire	−1.031	−1.628, 0.434	0.001
Lothian	−1.519	−2.029, −1.009	<0.001
Tayside	−0.784	−1.413, 0.155	0.015

*Lower SEP : Lower socioeconomic position (Scottish Standard Grade qualifications and below), which is also the reference category

**Higher SEP: Higher socioeconomic position (Scottish Higher Grade qualifications and above)

### Sensitivity analysis

When using clinical cut-offs (rather than quartiles for the operationalisation of allostatic load), those with higher SEP continued to show lower allostatic load after adjusting for age (*b* = −0.359, 95 % CI −0.501, −0.218, *p* < 0.001), equivalent to 7.3 years difference in physiological burden. As before, there was no effect modification from any of the contextual factors, although looking at the SEP patterning stratified by age showed a similar pattern to before, with inequalities becoming significant from ages 35 and up, before narrowing at older ages. The results stratified by sex, urban/rural and location mirrored those of the quartile-based allostatic load analyses (results not presented).

## Discussion

This study has explored in further detail the relationship between lower SEP and higher allostatic load across the lifecourse using a large, nationally representative population sample, finding that inequalities widen with age, before narrowing in later life through stratified analysis (although there was no statistically significant interaction effect between age and SEP). The social patterning of allostatic load appears to be consistent with sex, while there are more mixed findings with regards place (urbanity or geographical location).

While other studies have typically adjust for age or sex, the small number of studies that have considered the more nuanced patterns of inequalities in allostatic load have also shown some similar results. Geronimus (2006) showed inequalities in higher risk allostatic load (using poverty:income ratio) increase with age [18]. This study used data from the US National Health and Nutrition Examination Survey (NHANES IV, 1999–2002), but did not look at ages above 64 and focused on a binary measure considering the risk of having an allostatic load score of 4 or more. Additional analyses of the NHANES study (using NHANES III) has shown that these inequalities in allostatic load occur across adult life (ages 20-80+), also weakening with older age [47]. In analyses of socioeconomic inequalities using lifecourse measures of SEP (with allostatic load measured at age 43), Gustafsson et al. (2010) found that the inequalities were similar in men and women (although had potentially different mediators) [20]. Analyses of place effects on the relationship between SEP and allostatic load have typically focused on using neighbourhood measures of SEP (showing a potentially independent effect over and above individual SEP) [38]. However there is no other known evidence for place effects in terms of urbanity or geographic location.

Widening socioeconomic inequalities with age have been seen with various morbidities and mortality [48–50], including a typical pattern of these inequalities narrowing at older ages [51–54]. However, evidence from longitudinal studies has begun to find that these narrowing inequalities may be more of an artefact given survival bias (those with lowest SEP have the poorest health and therefore become underrepresented at older ages) and through using less proximal SEP measures [55]. The narrow inequalities at younger ages are also theorised to be due to the accumulated nature of disadvantage (and allostatic load) requiring time to show physiological and health consequences, with various potential mediators [22, 56]. Other studies have also found that the effect of SEP on health, rather than physiological dysregulation *per se*, increases with younger birth cohorts [57]. It has been suggested that this is the result of the changing contexts for the SEP-health association. For example, life expectancy has increased with younger cohorts; the differences in the meaning of different SEP measures have changed for different cohorts (e.g. the growing importance of education in people’s lives with younger birth cohorts); and the pattern of diseases has also altered across cohorts. While it is not possible in this study to unravel age and cohort effects, the possibility that there are cohort effects, rather than age effects present, may be a factor to consider. Given that our allostatic load measure contained pre- and post-clinical cut-offs, it is also telling that we witness the same patterns as seen with other morbidities and mortalities. However, the lack of statistical significance in the effect moderation analysis (compared to stratified analysis) suggests that the inequality seen at different ages is not as strong as perhaps seen with other more clinical outcomes. However, there remains a case for measures such as allostatic load to be considered as pre-clinical markers of ill health, and potential alternative outcomes/predictors when assessing health inequalities and the effects of interventions/programmes to improve health and reduce inequalities. What remains less known is if the allostatic load concept accurately predicts disease/death across the lifecourse (there is evidence that it does at older ages [13, 58, 59]) and if it offers any additional power over using single biomarkers or biomarkers across single physiological systems.

In this study we also considered the importance of place in the social patterning of allostatic load. There were no significant interaction effects, with allostatic load typically higher in lower versus higher SEP groups across all ten areas and urban/rural settings (although not always statistically significant). In terms of urbanity, it was not simply a case that the inequalities were present in the larger, more urban areas. Smaller sample sizes were present in the areas that were statistically non-significant, reducing the statistical power available. A similar inconsistent pattern was also seen with geographical location, where it was not the case that the areas with larger cities displayed the widest and significant inequalities. While the areas housing the major cities of Glasgow and Edinburgh (Greater Glasgow and Clyde and Lothian, respectively) did show significant inequalities, Grampian (Aberdeen city) and Tayside (Dundee city) did not. We did not consider area-level deprivation in this study as we viewed it a separate research question (does area-level SEP remain after adjusting for individual SEP), but it is possible that these larger area measures are less relevant to people’s health compared to more local-level measures (such as those derived from postcodes). In those studies that have considered area-level deprivation, it does appear there is an independent effect over and above individual SEP [21, 38].

As a sensitivity measure, we did consider the clinical cut-off operationalisation of allostatic load, finding that it mirrored the patterns seen in the ‘quartile’ distribution score. While arguments remain regarding the best operationalisation of allostatic load, the use of the distribution score measure is perhaps the most common [11]. This score is typically favoured given it matches the original allostatic load concept of a ‘sub-clinical dysregulation state’ more closely than a clinical-cut off score [11, 60]. However, it makes cross-sample comparisons more difficult as the cut-off points vary from one population to another. Clinical cut-offs do offer greater comparability though, important when considering the results of allostatic load studies (and perhaps more importantly, testing interventions involving allostatic load) in different populations and settings. However, it is relatively easy to measure allostatic load in more than one way to improve the reliability and comparability of results.

This study used a large, nationally representative sample of the Scottish population, a country with high-profile health inequalities compared to other regions of the UK and Europe [26, 27, 61]. This study also builds on the limited evidence that specifically focuses on how the socioeconomic patterning of allostatic load may differ given other demographic/contextual factors. Despite these strengths, we must note some potential limitations. First, the sample was cross-sectional in nature and would be improved upon by including a longitudinal component, especially in the progression of allostatic load over time. In addition, we have only included one population sample in this study. This study of the Scottish population may not be generalizable to other populations and, given the high allostatic load cut-offs (sometimes above clinical cut-offs), may also further highlight Scotland’s poor health record [62]. Third, although the overall sample size of the study was large, within some groupings small sample sizes were inevitable (e.g. small areas or locations). Looking at effect moderation using interaction terms revealed that the differences seen in the stratified analysis might be influenced by such small sample sizes (present in the youngest and oldest ages). A related issue is that of missing data, especially given the need for all 10 biomarkers being required to produce the allostatic load score. As well as reducing the sample size, there is the potential for the analysis sample to be substantially different to the original sample. However, comparisons between the whole ‘blood’ sample and the analysis sample for age, sex, urbanity, location and SEP revealed no substantive differences in frequencies (unpublished). The only category that perhaps showed underrepresentation was those age 75+ with lower SEP. The reasons for this were typically invalid/low blood samples or missing results from at least one of the anthropometric or blood measures. This may be relevant to the issues of survival bias discussed above with regards narrowing inequalities at these older ages. All analyses were weighted to be representative of the Scottish population using weights to correct for different selection probabilities and for non-response. However, while these weights make the ‘blood’ and ‘main’ sample comparable to the population in question, they cannot address issues of item missingness from respondents who did give samples.

Our allostatic load construct did not contain any markers from the hypothalamic pituitary adrenal (HPA) axis that forms part of the neuroendocrine system (stress response). The stress response is believed to play a key role in allostasis and subsequent allostatic load, with a cascade of events that starts with primary stress mediators, such as cortisol, before initial stress responses (‘primary effects’ such as rapid increases in blood pressure and sugars/fats that supply the body with extra energy) and then to secondary and tertiary outcomes (measured in our allostatic load model). These stress markers can be difficult to measure in large surveys where direct examination of the stress response (e.g. measuring cortisol) is difficult due to the circadian rhythms shown in these stress hormones (e.g. cortisol and DHEA-S) and the rapid sampling required in order to measure baseline versus activated levels (e.g. cortisol). This has made inclusion of these measures in large surveys less common than traditional clinical markers such as blood pressure or cholesterol. However, inclusion of measures such as cortisol or DHEA-S could improve the power of allostatic load as an earlier risk predictor for disease, but their exclusion does not invalidate this allostatic load construct as the subsequent outcomes of cortisol release are still being included. Finally there are potential limitations in the operationalisation of both allostatic load and SEP, and the subsequent interpretations that can be made (discussed above). We did perform sensitivity analysis with an alternative allostatic load construct and found no substantive differences. Although there remains no firm agreement on the best operationalisation of allostatic load, the quartile construct remains commonly used and relatively easy to interpret (compared to z-scores or more complex methods such as canonical correlation) [12, 63]. We did carry out sensitivity work using an alternative education cut-off (low SEP = no qualifications) to take into account changing educational attainment between the younger and older cohorts (not published). However, this measure produced a relatively small lower SEP group (emphasised in some stratified analyses) and meant the higher SEP group contained a broader grouping of educational attainment levels that may not best represent what ‘higher’ SEP is attempting to measure. Hence, the magnitude of the inequality and the statistical significance was reduced, despite demonstrating the same patterns. It could be possible to use other measures of SEP in addition to education, although in this sample this would have resulted in greater levels of missing data (e.g. using occupational-based social class, which also suffers from different meanings at different stages of the lifecourse). Education is “an indicator of SEP at the onset of adult life that sets an individual’s socioeconomic trajectory for the future” [64]. If follows that effects of SEP on cumulative physiological burden (such as allostatic load) may take many years to accumulate, so education may provide a more robust marker of SEP through, in particular, early adult life and middle age, compared to measures simply taken at the time of the sampling (e.g. housing status or occupational status/social class).

## Conclusions

This study has found that socioeconomic inequalities (measured using education) in allostatic load are present in a large, nationally representative study. These inequalities widen with age, before narrowing in later life, although there was no significant interaction effect between age and SEP. These findings are important in the case being made for allostatic load to be considered a pre-clinical risk marker that may help explain some of the mechanisms linking SEP and health. However, individuals’ demographic and contextual circumstances can play a role in the precise nature of this socioeconomic patterning and thereby alter the assumptions and effects of subsequent interventions to reduce such health inequalities. In addition, this evidence is important in our understanding of how socioeconomic factors can influence health across the lifecourse, not just in individual physiological systems, but throughout the body.

## Additional file

Additional file 1:
**Allostatic Load clinical cut-offs.** (DOCX 31 kb)
