# Supplementary material for: The importance of age, sex and place in understanding socioeconomic inequalities in allostatic load: Evidence from the Scottish Health Survey (2008–2011)
Source: BMC Public Health. 2016 Feb 9;16:126. doi: 10.1186/s12889-016-2796-4 (PMC4746832; doi:10.1186/s12889-016-2796-4)
Supplement: Additional file 1: — Allostatic Load clinical cut-offs. (DOCX 31 kb) [file 12889_2016_2796_MOESM1_ESM.docx]

**Web Table 1** Educational disparities in allostatic load (using clinical cut-offs), adjusted for age, sex, urban/rural and location

|  | **Beta (Lower* vs Higher** SEP)** | **95% Confidence Intervals** | **P-value** |
| --- | --- | --- | --- |
| Baseline (Higher SEP vs Lower SEP)* | -0.765 | -0.929, -0.602 | <0.001 |
| + Age | -0.416 | -0.561, -0.271 | <0.001 |
| + Age + Age^2^ | -0.565 | -0.725, -0.406 | <0.001 |
| + Age + Age^2^ +Age^3^ | -0.469 | -0.622, -0.317 | <0.001 |
| + Sex | -0.757 | -919, -0.594 | <0.001 |
| + Urban/Rural | -0.743 | -0.906, -0.580 | <0.001 |
| + Location | -0.790 | -0.950, -0.631 | <0.001 |
| + Age, Sex, Urban/Rural and Location | -0.434 | -0.578, -0.291 | <0.001 |

*Lower SEP : Lower socioeconomic position (Scottish Standard Grade qualifications and below), which is also the reference category

** Higher SEP: Higher socioeconomic position (Scottish Higher Grade qualifications and above)

**Web Table 2** Educational disparities in allostatic load (using clinical cut-offs), stratified by sex, age groups, urban/rural and geographic location

| **Covariate** | **Beta**  **(Lower* vs Higher** SEP)** | **95% Confidence Intervals** | **P-value** |
| --- | --- | --- | --- |
| **OVERALL EFFECT (Unadjusted)** | -0.765 | -0.929, -0.602 | <0.001 |
| **SEX** |  |  |  |
| Male | -0.452 | -0.682, -0.222 | <0.001 |
| Female | -0.360 | -0.541, -0.180 | <0.001 |
| **AGE** |  |  |  |
| 18-24 | -0.159 | -0.590, 0.273 | 0.467 |
| 25-34 | -0.210 | -0.670, 0.268 | 0.400 |
| 35-44 | -0.443 | -0.751, -0.135 | 0.005 |
| 45-54 | -0.536 | -0.886, -0.186 | 0.003 |
| 55-64 | -0.421 | -0.724, -0.119 | 0.006 |
| 65-74 | -0.456 | -0.871, -0.040 | 0.032 |
| 75+ | -0.496 | -1.054, -0.061 | 0.080 |
| **URBAN/RURAL** |  |  |  |
| Primary City (pop>125,000) | -0.424 | -0.664, -0.184 | 0.001 |
| Urban (pop>10,000) | -0.441 | -0.726, -0.155 | 0.003 |
| Small Accessible town (pop>3,000) | -0.259 | -0.707, 0.190 | 0.256 |
| Small Remote town (pop>3,000) | -0.350 | -0.804, 0.104 | 0.127 |
| Accessible Rural | -0.832 | -1.194, -0.470 | <0.001 |
| Remote Rural | -0.314 | -0.171, 0.798 | 0.202 |
| **LOCATION** |  |  |  |
| Ayrshire & Arran | -0.249 | -0.752, 0.253 | 0.324 |
| Borders, Dumfries & Galloway | -0.130 | -0.728, 0.468 | 0.665 |
| Fife | -0.604 | -1.250, 0.042 | 0.066 |
| Forth Valley | -0.783 | -1.503, -0.063 | 0.034 |
| Grampian | -0.245 | -0.713, 0.223 | 0.302 |
| Greater Glasgow & Clyde | -0.469 | -0.758, -0.181 | 0.002 |
| Highland & Islands | -0.254 | -0.670, 0.161 | 0.226 |
| Lanarkshire | -0.469 | -0.939, 0.001 | 0.050 |
| Lothian | -0.709 | -1.081, -0.336 | <0.001 |
| Tayside | -0.200 | -0.633, 0.234 | 0.363 |
|  |  |  |  |

*Lower SEP : Lower socioeconomic position (Scottish Standard Grade qualifications and below)

** Higher SEP: Higher socioeconomic position (Scottish Higher Grade qualifications and above)
